# Hospital Managers and Probationer's Contracts

**Published:** 1911-11-18

**Authors:** 


### HOSPITAL MANAGERS AND PROBATIONER'S CONTRACTS.

The usual system which regulates the mutual obligations of a hospital and a probationer nurse is a tacit acknowledgment of the benefits which each legitimately derives from the other. The essence of the system is that the hospital provides for the probationer board and lodging, and certain other advantages, of which medical attendance is an important one; and a course of instruction, theoretical and practical, in the art of sick nursing. The hospital certificate that the nurse has duly profited by it, is a definite, tangible, and marketable commodity, and as such is of value to its possessor. Incidentally it may be noted that the higher the reputation of the hospital and of its nursing standards and efficiency, the greater is the value of its certificate and the higher the salaries, and therefore the more exacting may fairly be the requirements from the nurse.

On the other side, the hospital demands from the nurse her labour; at first this labour is unskilled, but gradually, as training goes on, becomes more and more efficient in course of time. This labour is arduous in the extreme, more arduous than is recognised by any except those whose opportunities for knowing are intimate and constant. A strict medical examination before acceptance ensures that none but specially robust women enter upon this exacting work, and even then the percentage of physical breakdowns is high.

Now the period probably during which the probationer profits the most from her training is her first year; and the period at which the hospital profits most from the probationer's labour is her last year. It is evident, therefore, that when a probationer is released from her engagement midway through her period of training, she has in fact the best of the bargain. Assuming that this bargain has been originally a fair one, it is not unjust that the probationer desiring release from her duties before the probation period has expired should contribute a sum of money in lieu of that expert service of which she is depriving the hospital. So it comes about that in the binding form of contract which is signed by the probationer when she enters on her training, provision is often made for a fine or a graduated system of fines should the contract be broken by the nurse for reasons other than ill-health. That such a provision should be inserted is, as has been shown, fair in principle and necessary for the protection of the hospital against sudden desertions due to passing whims or emotions. It operates mainly as a prevention of ill-considered resignations. It is inconceivable that it should be twisted into an instrument of extortion or oppression; for those who put it to such purposes would ill deserve the confidence of the public or of nurses, and bring the sacred name and cause of charity into odium and contempt.

We make these remarks with the more conviction and emphasis because an instance has been brought to our notice in which the whole principle underlying this necessary clause in probationers' contracts seems on the face of it to have been abused. At one of the great general hospitals in England probationer nurses sign a contract for four years' service. In that document it is laid down that on voluntary resignation they must pay a "fine" of £10, and that this is not of the nature of "liquidated damages." The meaning of this phraseology, which is slightly cryptic to non-legal minds, is shown very clearly by the incident we refer to. A particularly bright and capable woman became a probationer at the institution in question. At the end of two years' training she became staff nurse in charge of a ward, under the supervision of a sister who had two wards to control. It is thus proved that her aptitude was marked. She remained in this position for another year, at the end of which she requested permission to leave for the purpose of marrying. The authorities of the hospital where this nurse was training informed her that the interpretation of her signed contract was that she would have to pay, for the privilege of resigning, not only the £10 fine, but also a further £70 as "liquidated damages" for the loss of her services for a year! The nurse and her fiancé resisted this outrageous demand, and were then confronted with legal proceedings for the recovery of the £80. These proceedings were compromised out of court for the sum of £20: this shows how weak was the case for the hospital authorities, or rather how impossible it was to take it into the publicity of the courts. Such arbitrary acts carry their own condemnation with them.

